# CXCR7 expression in esophageal cancer

**DOI:** 10.1186/1479-5876-11-238

**Published:** 2013-09-30

**Authors:** Michael Tachezy, Hilke Zander, Florian Gebauer, Katharina von Loga, Klaus Pantel, Jakob R Izbicki, Maximilian Bockhorn

**Affiliations:** 1 Department of General, Visceral and Thoracic Surgery, University Medical Center Hamburg-Eppendorf, Martinistrasse 52, 20246 Hamburg, Germany; 2 Department of Pathology, University Medical Center Hamburg-Eppendorf, Hamburg, Germany; 3 Institute of Tumor Biology, University Medical Center Hamburg-Eppendorf, Martinistrasse 52, 20246 Hamburg, Germany

**Keywords:** CXCR4, CXCR7, CXCL12, Esophagus, Esophageal cancer

## Abstract

**Background:**

The chemokine CXCL12 and its receptor CXCR4 play a major role in tumor invasion, proliferation and metastasis in different malignant diseases, including esophageal carcinoma, amongst others. CXCR7 was recently identified as a novel alternate receptor for CXCL12. The aim of this study was to evaluate the prognostic impact of expression of chemokine receptor CXCR7 in patients with esophageal carcinoma (EC).

**Methods:**

Expression of CXCR7 in primary tumors, lymph nodes and distant metastases of 299 patients with EC was evaluated by immunohistochemistry on a tissue microarray and compared with clinical and histopathological data.

**Results:**

In esophageal cancer sections, CXCR7-specific reactivity was apparent in 45% of the squamous cell carcinomas (ESCC), but only occasionally in adenocarcinomas. No correlation between CXCR4 and CXCR7 expression was evident. We correlated expression with clinical and histopathological characteristics, but could not find any association.

**Conclusions:**

Contrary to the other known CXCL12 receptor, CXCR4, CXCR7 is expressed in ESCC only, underlining the divergent mechanisms and backgrounds of EAC and ESCC. The results of the study do not indicate a significant functional role for CXCR7 in EAC or ESCC of the esophagus. However, its variable expression in the main two main types of EC needs to be further investigated.

## Background

Esophageal carcinoma (EC) is one of the most aggressive solid tumors and has an enormous malignant potential for local invasion and early dissemination, resulting in a high rate of tumor recurrence after surgical treatment that is intended to be curative [1-3]. Any effects of multimodal therapeutic approaches such as neoadjuvant radiochemotherapy on survival and quality of life seem to be modest and are therefore not generally accepted [4,5]. Surgery remains the treatment of choice for resectable EC [6,7]. Biologic, targeted therapies have not found their way into the clinical routine yet, although some promising targets have been identified [8].

Previous reports have shown a crucial role of chemokines and their receptors in tumor growth, angiogenesis, and tumor cell homing in lymph nodes, bone marrow and distant metastases [9-11]. In particular, the chemokine CXCL12 (SDF-1) is a broadly expressed cytokine that plays important roles in embryogenesis, hematopoiesis and tumorigenesis of various entities [12,13]. At present, two receptors of SDF-1 α have been identified, CXCR4 and CXCR7, which may act as contributing factors in metastasic spread of melanoma, breast, gastric, and EC [12,14]. While several authors have investigated CXCR4 expression, its function and prognostic impact in EC, no data are available about the expression of CXCR7 in EC [3,15-20]. Inhibition of the CXCR4 receptor by specific antagonists has shown an anti-proliferative effect in EC and other entities in vitro and in vivo in xenograft mouse models [13,15,16,21]. Phase 1 trials are investigating the inhibitory potential of CXCR4 antagonists in vivo in several other entities (for example high-grade glioma; NCT01339039 or multiple myeloma; NCT00903968).

Since CXCR7 is the other known receptor for SDF-1 α, the question must be raised whether CXCR7 has a similar function as CXCR4 in the tumor cell biology of EC. Therefore, the aim of this study was to investigate the expression of CXCR7 in a large number of EC specimens and to evaluate its potential role as a prognostic and therapeutic target molecule.

## Patients and methods

### Study design and patients

For this study, 299 patients with EC who underwent surgery at University Medical Center Hamburg-Eppendorf between February 1992 and May 2005 were chosen according to available tumor material. None of the patients received neoadjuvant or adjuvant treatment. All data including sex, histology, depth of tumor invasion, lymph node metastasis, tumor type, disease stage (UICC 7th edition) and routinely performed screening for DTC in bone marrow were obtained from a combination of clinical and pathological record review, outpatient clinic medical records and communication with patients and their attending physicians [22].

This study was approved by the ethics committee of the chamber of physicians, Hamburg, Germany. Written informed consent was obtained from all patients to use their resected tumor.

### Tissue microarray (TMA)

The pre-existing tissue microarray consisted of 299 primary lesions and corresponding lymph nodes (n=147) and distant metastases (n=46) from patients with EC, together with 10 specimens of healthy esophagus mucosa [23,24]. After surgical resection, the specimens were fixed in 4% buffered formalin, paraffin embedded, and used for TMA construction as previously described [23]. Briefly, hematoxylin-eosin stained sections were made from selected primary tumor blocks (donor blocks) to define representative tumor regions. Tissue cylinders (0.6 mm in diameter) were then punched from that region of the donor block using a homemade semi-automated tissue arrayer. Three-micron sections were cut using the Paraffin Sectioning Aid System (Instrumentics, USA).

### Immunohistochemistry and scoring

The CXCR7 staining protocol was optimized in an extensive and standardized multi-step procedure on various benign and malignant tissues; the protocol was modified until selective staining with the lowest background signals was established [25]. Freshly cut TMA sections were analyzed on one day in a single experiment. After deparaffination and drying overnight at 37°C, sections were rehydrated in Tris-buffered saline (TBS = 0.05 M Tris–HCl at pH 7.6 and 0.15 M NaCl). CXCR7 pre-treatment was performed with target retrieval solution (Dako) at 121°C for 5 minutes. Sections were blocked with human AB serum (Biotest Diagnostics, Dreirach, Germany) diluted 1:10 in TBS for 20 minutes. Primary CXCR7 antibody was used at a 1:150 dilution (IgG2a, Clone 358426, R&D Systems) overnight at 4°C. A standard indirect immunoperoxidase procedure was used for visualization of bound antibody (Envision System, DAKO). Diaminobenzidine was used as the chromogen. Sections were counterstained with hematoxylin solution (Merck, Darmstadt, Germany).

Complete TMA sections were scanned with a Zeiss MIRAX (Carl Zeiss MicroImaging GmbH, Göttingen, Germany) digital high-resolution slide scanner and reviewed using MIRAX Viewer Version 1.12 for Windows XP (Carl Zeiss MircoImaging).

The staining intensity (0, 1+, 2+, 3+) and the fraction of positive tumor cells were scored for each tissue spot as recently published [25]. Spots without staining and spots with a staining intensity of 1+ in ≤20% of tumor cells were classified as CXCR7 negative. Spots with a staining intensity of 1+ in >20% tumor cells, and spots with staining intensity ≥2 were classified as CXCR7 positive.

Immunohistochemical analysis of the sections was performed without knowledge of the patients’ identity or clinical status.

### Statistical analysis

SPSS Statistics for Windows (Version 17, SPSS Inc., Chicago, IL USA) was used for statistical analysis. Relationships between the immunostaining and clinicopathological data were calculated using Chi-square and Fisher’s-Exact tests and displayed in cross tables. Group Survival curves were plotted using the Kaplan-Meier method and analyzed by log-rank test. All tests were two-sided and p-values less than 0.05 were considered statistically significant.

## Results

### Characteristics of the patients

Tissue specimens of 299 patients aged 35 to 93 years (median 63 years) with the diagnosis of EAC (45%) and ESCC (55%) were included in this study. Of these, 235 men (79%) and 64 women (21%) were treated surgically between 1992 and 2005 in the Department of General, Visceral and Thoracic Surgery of the University Medical Center Hamburg-Eppendorf, Germany. Operation methods were transhiatal or abdominothoracic (Ivor Lewis) esophagectomy with intrathoracic or cervical anastomosis. Histopathologic findings are summarized in Table 1. Bone marrow aspirates of 190 patients were analyzed and disseminated tumor cells were identified in 34% of the patients (n=65). Median follow-up time of all patients included in the survival analysis was 15 months (range 0–178 months); calculated median OS for all patients was 15.0 months. Twenty-two patients (7.4%) died within the first 30 days after surgery.

**Table 1:** Correlation between CXCR7 expression and histopathologic cell type in primary tumors (PT), lymph node (LN) and distant metastases (Met)

			**Histopathologic cell type**		
		**Total**	**ESCC**	**EAC**	**p-value**
CXCR7 status					
PT	Negative	215	87 (55%)	128 (98%)	
	Positive	75	72 (45%)	3 (2%)	0.000
LN	Negative	96	33 (60%)	63 (93%)	
	Positive	27	22 (40%)	5 (7%)	0.000
Met	Negative	33	6 (43%)	27 (96%)	
	Positive	9	8 (57%)	1 (4%)	0.000

### CXCR7 Expression in primary tumors, lymph nodes and distant metastases of ESC and EAC

**Figure 1:**
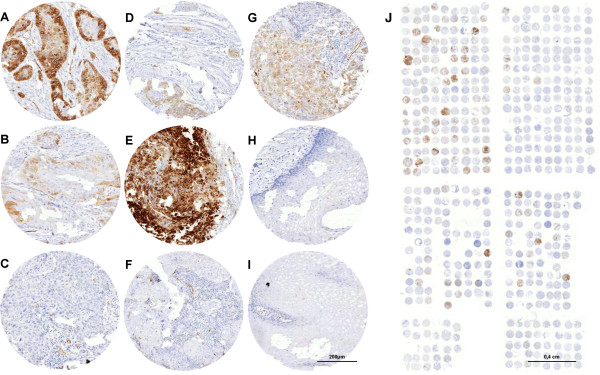
**CXCR7 immunohistochemistry.** Representative immunohistochemical CXCR7 staining of primary lesions of ESCC of the esophagus **(A)** strongly positive, **(B)** weakly positive and **(C)** negative. **(D)** Primary lesion of a CXCR7-positive EAC, **(E)** CXCR7-positive lymph node metastasis, **(F)** CXCR7-negative lymph node metastasis and **(G)** positive liver metastasis. **(H)** and **(I)** Healthy esophageal mucosa. **(J)** Complete scan of the esophagus TMA.

A total of 290 (97%) primary EC tumor samples were interpretable in our tissue microarray (TMA) analysis. Reasons for non-informative cases (n=9; 3%) included a complete lack of tissue samples or the absence of unequivocal cancer tissue in the TMA sections. Immunohistochemical staining for CXCR7 showed cytoplasmic expression of the molecule with a membranous accentuation. CXCR7 was not found in healthy esophageal mucosa cells, but only in endothelial cells (n=10, Figure 1H and 1I). The morphologic staining pattern did not differ between EAC and ESCC; both tumors showed a heterogeneous staining pattern inside the cancerous lesions (Figure 1A-G).

Only a small subset of EAC primary lesions (2%), lymph nodes (7%) and distant metastases (4%) were CXCR7 positive, while nearly half of the primary ESCC expressed CXCR7 (45%). According to this, CXCR7 expression is present significantly more often in ESCC than in EAC tumors (p<0.001). Of the ESCC lymph node metastases, 40% were CXCR7 positive, as well as 57% of the distant metastases (Table 1 and Figure 1A-1G).

### Correlation of CXCR7 expression in ESCC patients with clinical and histopathological characteristics

Due to the fact that CXCR7 is expressed in ESCC only, no statistical analysis is presented for EAC patients. As shown in Table 1, statistical analysis of the immunohistological staining results revealed no association with the tested characteristics (age, sex, disease stage (T, N, M status), tumor grading (G), LN or Met CXCR7 status). Results of CXCR7 immunohistochemistry were correlated with the recently published data regarding CXCR4 expression [3]. In total, 95 specimens were stained for both receptors; Forty-six patients with ESCC and 49 with EACC. Only one of the CXCR4 stained EACC specimens was CXRC7 positive, not enough for statistical analysis. No significant correlation was seen between CXCR4 and CXCR7 expression in ESCC (p=0.128, Table 2).

**Table 2:** Correlation between CXCR7 expression and clinicopathological data

			**Total**	**CXCR7 primary tumor (ESCC)**				**p-value**
				**CXCR7 negative**		**CXCR7 positive**		
				**(n=87, 55%)**		**(n=72, 45%)**		
Median Age, years (range)			159	60.9	(36–80)	62.1	(37–93)	0.158
Sex								
	Female		119	70	(59%)	49	(41%)	
	Male		40	17	(43%)	23	(57%)	0.098
Disease Stage (UICC 7^th^ edition)								
	T	1	23	15	(65%)	8	(35%)	
		2	33	18	(55%)	15	(45%)	
		3	91	47	(52%)	44	(48%)	
		4	12	7	(58%)	5	(42%)	0.698
	N	0	67	36	(54%)	31	(46%)	
		1-3	92	51	(55%)	41	(45%)	0.873
	M	0	158	86	(54%)	72	(46%)	
		1	1	1	(100%)	0	(0%)	1.000
	G	1	4	2	(50%)	2	(50%)	
		2	113	60	(53%)	53	(47%)	
		3	42	25	(59%)	17	(41%)	0.761
Prognostic Tumor Staging (UICC 7^th^ edition)								
	Ia°		19	12	(63%)	7	(37%)	
	Ib°		16	8	(50%)	8	(50%)	
	IIa°		28	14	(50%)	14	(50%)	
	IIb°		13	8	(61%)	5	(39%)	
	IIIa°		40	21	(52%)	19	(47%)	
	IIIb°		20	10	(50%)	10	(50%)	
	IIIc°		22	13	(59%)	9	(41%)	
	IV°		1	1	(100%)	0	(0%)	0.931
Bone marrow micrometastasis								
	Negative		61	34	(56%)	27	(44%)	
	Positive		34	17	(50%)	17	(50%)	0.670
CXCR7 status								
	LN	Negative	32	17	(53%)	15	(47%)	
		Positive	20	9	(45%)	11	(55%)	0.776
	Met	Negative	6	4	(67%)	2	(33%)	
		Positive	8	4	(50%)	4	(50%)	0.627
CXCR4 status								
	PT	Negative	17	12	(71%)	5	(29%)	
		Positive	29	13	(45%)	16	(55%)	0.128

**Figure 2:**
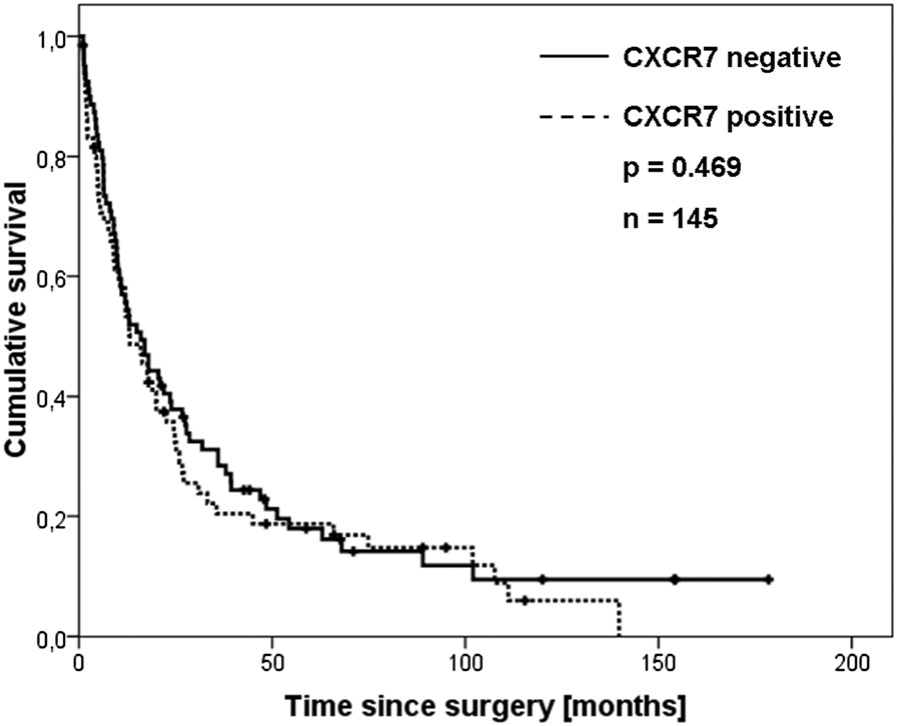
**Kaplan-Meier overall-survival analysis for patients suffering from ESCC.** Groups with and without CXCR7 expression in ESCC were compared. P-values were calculated with the log-rank test.

Overall survival curves plotted by Kaplan-Meier analysis revealed no significant difference between CXCR7 negative and positive patients (p=0.469, Figure 2).

## Discussion

The CXCL12/CXCR4/CXCR7 chemokine axis has been identified as an important element in tumor development, progress and metastasis [13]. In EC, several authors have investigated the expression and function of CXCL12 and CXCR4 and found pro-proliferative and metastatic potential as well as a significant association with shortened OS, but no studies regarding the expression, prognostic significance or function of CXCR7 in EC have been published [3,15-20]. The current study presents the results of an immunohistochemical CXCR7 staining experiment on primary and metastatic specimens in a large cohort of EC patients.

In normal esophageal mucosa, no expression was found except in endothelial cells [26]. CXCR7 expression was rarely found in EAC (2%), but was over-expressed in almost half of the ESCC (45%). A similar phenomenon has been described for non-small lung cell cancer (NSCLC); CXCR7 is expressed predominantly in squamous cell carcinoma and occasionally in adenocarcinoma [27]. In contrast to this, the published studies investigating CXCR4 expression have shown a similar rate in EAC and ESCC, which might be another hint as to their divergent functional roles in EC [3,15,17]. A recently published study of our group has immunohistochemically analyzed the CXCR4 expression of esophageal cancer specimen on conventional histological slides. Ninety five of them were implemented in the CXCR7 stained TMA and analyzable. The expression analysis of both receptors revealed a statistically co-expression neither in ESCC nor in EAC. This discrepant presentation has already been shown in an expression analysis study of lung cancer specimen [28]. Based on their results, Imai and colleagues suggested that CXCL12 may differentially interact with CXCR4 or CXCR7, depending on the cellular context [28]. The molecular mechanisms of CXCR7 are poorly understood and so are its physiologic and oncologic functions in tumor development and progression, whereas a pro-proliferative role of CXCR7 for tumor cells and neovascularisation has been described [13,26]. CXCR7 is described as a potential, but according the results of our study not essential modulator or co-receptor for CXCR4 signaling. The infrequent expression of CXCR7 in EAC might explain why CXCR4 inhibitors have shown, due to the absence of the alternate receptor CXCR7, a significant effect in EAC in in-vitro and in-vivo experiments [16,21]. Similarly, if CXCR7 might function as an escape receptor for CXCR4, merely inhibiting CXCR4 expression might have no significant effect in ESCC.

In general, the significantly different expression rate in EAC and ESCC underlines the biologic divergence between the two types of EC tumors, which were assumed to behave in a similar fashion for a long time [29,30]. As a consequence, the recently published, 7th AJCC/UICC classification has proposed different prognostic staging groups for EAC and ESCC [31].

Contrary to other entities, such as breast, prostate and lung cancer, neither was an association seen between CXCR7 expression in primary lesions and clinical and histopathological data, nor was it shown to have an impact as a prognostic marker for survival of patients with ESCC [27,32,33].

## Conclusions

In conclusion, the results of the study do not indicate a significant functional role for CXCR7 in EC. Nevertheless, CXCR7 is detectable in a high number of ESCC patients and since biologic functions have been reported in recent literature, it might serve as a possible target for molecular therapies.

However, the functional background for its divergent expression in EAC and ESCC and the functions and interactions in EC between CXCR7, its ligand SDF-1 α and the other members of the cytokine family should be further investigated.

## Competing interests

The authors declare that they have no competing interests.

## Authors’ contributions

MT conceived the study and participated in its design and coordination, drafted the manuscript, performed the statistical analysis and collected the clinicopathological data. FG conceived the study and participated in its design and coordination and collected the clinicopathological data. HZ conceived the study and participated in its design and coordination and drafted the manuscript. MB performed the statistical analysis and participated in the design of the study and coordination and helped to draft the manuscript. KL participated in the establishment of immunohistochemistry and analyzed the arrays. KP participated in the design of the study and coordination and helped to draft the manuscript. JRI participated in the study design and coordination and helped to draft the manuscript. All authors read and approved the final manuscript.
